# Riccardin D Exerts Its Antitumor Activity by Inducing DNA Damage in PC-3 Prostate Cancer Cells *In Vitro* and *In Vivo*

**DOI:** 10.1371/journal.pone.0074387

**Published:** 2013-09-17

**Authors:** Zhongyi Hu, Feng Kong, Manfei Si, Keli Tian, Lin Xi Yu, Charles Y. F. Young, Huiqing Yuan, Hongxiang Lou

**Affiliations:** 1 Department of Biochemistry and Molecular Biology, Shandong University School of Medicine, Jinan, China; 2 Department of Natural Product Chemistry, Shandong University School of Pharmaceutical Sciences, Jinan, China; 3 Department of Human Biology, University of Toronto, Toronto, Ontario, Canada; 4 Department of Urology, Mayo Clinic College of Medicine, Mayo Clinic, Rochester, Minnesota, United States of America; Texas Tech University Health Sciences Center, United States of America

## Abstract

We recently reported that Riccardin D (RD) was able to induce apoptosis by targeting Topo II. Here, we found that RD induced cell cycle arrest in G2/M phase in PC-3 cells, and caused remarkable DNA damage as evidenced by induction of γH2AX foci, micronuclei, and DNA fragmentation in Comet assay. Time kinetic and dose-dependent studies showed that ATM/Chk2 and ATR/Chk1 signaling pathways were sequentially activated in response to RD. Blockage of ATM/ATR signaling led to the attenuation of RD-induced γH2AX, and to the partial recovery of cell proliferation. Furthermore, RD exposure resulted in the inactivation of BRCA1, suppression of HR and NHEJ repair activity, and downregulation of the expressions and DNA-end binding activities of Ku70/86. Consistent with the observations, microarray data displayed that RD triggered the changes in genes responsible for cell proliferation, cell cycle, DNA damage and repair, and apoptosis. Administration of RD to xenograft mice reduced tumor growth, and coordinately caused alterations in the expression of genes involved in DNA damage and repair, along with cell apoptosis. Thus, this finding identified a novel mechanism by which RD affects DNA repair and acts as a DNA damage agent in prostate cancer.

## Introduction

Prostate cancer (PCa) is one of the most common malignant tumors in men and hormonal withdrawal therapy remains effective for advanced PCa. However, the development of hormone-refractory prostate cancer (HRPC) occurs inevitably after hormonal deprivation therapy [1,2]. There are limited options for the successful management of HRPC. Recently, docetaxel, a plant alkaloid derivative, has been emerging as an active agent to improve quality of life and survival conditions in patients with metastatic HRPC [3,4]. The success of docetaxel has led to many efforts being made to isolate various naturally occurring chemicals and to investigate mechanisms of action of bioactive compounds for the development of chemopreventive and/or therapeutic agents to treat cancers including HRPC [5].

One of the most efficient chemical reagents used in cancer chemotherapy are DNA damage inducers, which can cause a variety of DNA lesions via multiple mechanisms. For example, camptothecin and etoposide can trigger single-strand breaks (SSBs) or double-strand DNA breaks (DSBs) by trapping topoisomerase-DNA covalent complexes, subsequently leading to the cell death [6,7]. Thus, DNA topo I and II, especially topo II, are believed to be well-established targets in cancer therapy.

Depending on the type of DNA lesions, specific cell cycle checkpoints and cellular cascades are activated by DNA-damaging agents. As widely accepted, ataxia telangiectasia mutated (ATM) and ataxia telangiectasia and Rad3 related (ATR) signaling pathways play important roles in response to DNA damage. ATM responds mainly to DSBs, and initiates phosphorylation of downstream targets such as Chk2, BRCA1, and NBS1 proteins at the site of DNA damage [8]. These factors act together to induce G1, S, and G2 cell cycle arrests, DNA repair, and/or activation of cell death pathways [9]. While ATR is activated in response to replication stress, it triggers the activation of Chk1, which in turn leads to the phosphorylation of Cdc25 and prevents the activation of CDK1/Cyclin B and mitotic entry [10]. Upon DSBs, the process of DSBs end joining involves numerous proteins and enzymes through nonhomologous end joining (NHEJ) and homologous recombination (HR) repair mechanisms [11,12]. For example, the Ku70/86 heterodimer is critical in NHEJ, since it binds to the broken DNA ends and recruits repair-related proteins including DNA-dependent protein kinase, XRCC4, and DNA Ligase IV [13]. It has been demonstrated that DNA damage is implicated to elicit both ATM and ATR signaling [14]. Activation of these two pathways with possible defects in the cell cycle checkpoints and DNA repair response may be relevant in determining the potency and efficacy of DNA damage inducers.

We have recently reported that Riccardin D (RD), a macrocyclic bisbibenzyl compound from the Chinese liverwort plant *Dumortierahirsute* [15], was able to induce apoptosis of human leukemia cells by targeting topo II [16]. In this study, we found that RD treatment led to the induction of DNA damage and the inhibition of response products involved in DNA repair.

## Materials and Methods

### Cell culture and treatments

Human LNCaP, PC-3 and DU145 cells (The American Type Culture Collection (ATCC)) were cultured in RPMI 1640 medium (HyClone) supplemented with 10% fetal bovine serum (HyClone). The cells were cultured in 5% CO_2_ at 37°C until reaching approximately 50–70% confluence then treated with chemicals. RD was isolated and purified in our laboratories as described previously [15]. RD and Etoposide (VP-16) were prepared in dimethyl sulfoxide (DMSO) and stored as small aliquots at −20 °C.

### Immunoblotting

After treatment as indicated, cell lysates were prepared using RIPA buffer. Proteins (80 µg) were separated by SDS–PAGE and electrophoretically transferred onto polyvinylidene fluoride membranes (Millipore). The membranes were probed overnight at 4°C with the appropriate primary antibodies: glyceraldehyde-3-phosphate dehydrogenase (GAPDH), Cyclin E, poly (ADP-ribose) polymerase (PARP), Bcl-2, Bax and nucleolin (Santa Cruz), Ku70 and Ku86 (Active Modif), Cdc25B (BD Biosciences), Cyclin A (Anbo Biotechnology), Cyclin B1 (Novus Biologicals), Cdc25C, Ser^1981^-phosphorylated-ATM, Tyr^15^-phosphorylated-Cdc2, Ser^428^-phosphorylated-ATR, Ser^296^-phosphorylated-Chk1, Thr^68^-phosphorylated-Chk2, Ser^1524^-phosphorylated-BRCA1, Ser^139^-phosphorylated histone H2AX (γH2AX), PP2AA, PP2AB, and PP2AC (Cell Signaling), PPP4C (Bethyl, Montgomery, TX, USA), IgG-TRITC (Abcam) followed by blocking with 5% fat-free dry milk. Upon removal of primary antibodies, the membranes were washed with TBST and incubated with IgG-horseradish peroxidase (HRP)-conjugated secondary antibodies, then visualized by enhanced chemiluminescence detection system (Millipore).

### Cell growth and cell viability assay

Cells were seeded at 4000 cells/well in microplates (Roche), and exposed to RD or vehicle. Cell growth curves were obtained by the Real-Time Cell Analyzer SP Instrument (Roche). Cell Index (CI) values were normalized with respect to the CI value of the time point at which the chemical added. For cell viability analysis, PC-3 cells were pretreated with 10 mmol/L caffeine for 1 h, and exposed to RD or vehicle for 24h. Cell proliferation was then examined by 3-(4, 5- dimethylthiazol-2-yl)-2, 5-diphenyl-2H-tetrazolium bromide (MTT, Sigma) colorimetric assay.

### Cell cycle and apoptosis assay

After treatment with RD for 24h, cells were ﬁxed and treated with propidium iodide (PI) (Sigma) for 30-min in the dark. Cell cycle was analyzed by a FACS (Becton Dickinson, USA). Apoptosis was studied using an Annexin V-FITC ⁄ PI Apoptosis Detection Kit (BD Biosciences) by flow cytometry.

### Micronucleus assay

Cells were seeded in 6-well plates and treated with RD, DMSO or VP-16 (10 µmol/L) for 24h. After fixing and permeabilizing, cells were stained with DAPI (Sigma). Micronuclei in cells were scored under a phase-ﬂuorescence microscope (Nikon). At least 1000 cells per sample were scored for analysis.

### Neutral comet assay

Cells were treated with chemicals as indicated above. DNA DSBs were detected using the Trevigen Comet, Assay Single Cell Gel Electrophoresis Assay (Trevigen). Comet tails were imaged by a phase-fluorescence microscope (Nikon) and quantitated by Casp software. A minimum of 100 cells were scored per treatment.

### Immunoﬂuorescence staining of γH2AX and p-BRCA1

Cells grown on coverslips were fixed with ice-cold methanol/acetone (1:1) and incubated with 3% BSA in PBS with 0.1% Triton X-100. Following incubation with primary antibodies and rinsing with PBS, cells were immunostained with secondary antibodies and counterstained with DAPI. Fluorescence images were captured using a confocal microscopy (CarlZeiss).

### Microarray and real-time PCR analysis

Total RNA was extracted from cells that were exposed to chemicals using an RNAiso plus kit (Takara Bio). Microarray analysis was performed at Genetimes Technology, Inc. (China) on the Affymetrix HG-U133 plus 2 chip, and the original data was submitted to the GEO (Series GSE37812). For quantitative PCR (qPCR) assays, cDNA was synthesized using ReverTra Ace qPCR RT Kit (Toyobo). qPCR was performed with SYBR Green reaction master mix on a Real-time PCR System (Eppendorf International). Transcriptional levels of desired genes were normalized to the level of GAPDH. Changes in the gene expression were calculated using the ΔΔC_t_ method. The sequences of primers were shown in Table 1.

**Table 1:** Q-PCR forward and reverse primers.

	Forward primer (5'->3')	Reverse primer (5'->3')
Cdc25B	GGCTGAGGAACCTAAAGCCC	TCGATCTCATCGTGACACAGT
Cdc25C	AGGAACCCCAAAATGTTGCCT	GCAGAAGTGGTAAGCTGAGTG
cyclin E	TCCAAGAGTTTGCTTACGTCAC	GCCAGGAGATGATTGTTACAGG
cyclin A	GATGGTAGTTTTGAGTCACCACA	CACGAGGATAGCTCTCATACTGT
cyclin B1	ATAAGGCGAAGATCAACATGGC	TTTGTTACCAATGTCCCCAAGAG
cdc2	ACACAAAACTACAGGTCAAGTGG	GGAATCCTGCATAAGCACATCC
RPA1	CTCGGGAATGGGTTCTACTGT	CACTTGGACTGGTAAGGAGTGA
RPA2	TGGTAGCCTTTAAGATCATGCCC	CTGGATTGCTGATAGGTGCTC
RPA3	TGATGGAACCCCTTGATGAAGA	CATGTTGCACAATCCCTAAAGGA
XRCC5	GACGTGGGCTTTACCATGAGT	TCAGTGCCATCTGTACCAAAC
XRCC6	AGTCATATTACAAAACCGAGGGC	CCTTGGAGGCATCAACCAAAAA
MSH6	AGCTTAAAGGATCACGCCATC	AAGCACACAATAGGCTTTGCC
GAPDH	TGGTCACCAGGGCTGCTT	AGCTTCCCGTTCTCAGCCTT

### Co-immunoprecipitation

Aliquots of proteins (1.2 mg) from control and RD-treated cells were precleared with protein A/G Plus-Agarose (Santa Cruz) in the presence of non-specific IgG (Santa Cruz), then incubated with primary antibodies and 20 µl agarose beads with continuous mixing overnight at 4°C. The beads were washed and heated at 75°C for 5 min in loading buffer prior to immunoblotting assays.

### Analysis of DNA end-binding activity of Ku70/Ku86

Nuclear extracts from control or RD-treated cells were prepared, and subjected to the detection of DNA end-binding activity of Ku70 and Ku86 using a Ku70/Ku86 DNA Repair kit according to the manufacturer’s instructions (Active Motif).

### DNA end-joining assay

To determine the effects of RD on DSBs repair, we developed a cell-free DNA end-joining assay as described by Shao C, et al. [17]. The plasmid pUC19 was digested with HincII, resulting in a blunt-ended linear molecule. The linearized DNA was incubated with nuclear extract (2µg) in 50 mmol/L Tris-HCl (pH 7.6), 10 mmol/L MgCl_2_, 1mmol/L dithiothreitol, 1 mmol/L ATP, and 25% (w/v) polyethylene glycol 8000 at 14 °C for 6h or 24h. Nuclear extract of Raji cells purchased from Active Motif served as a positive control. The NHEJ was determined by PCR amplification of the junction of rejoined ends with M13 primers. PCR products of ampicillin were performed as an internal control. All experiments were repeated three times.

### NHEJ and HR repair assay

The ability of DNA repair in cells treated with RD was evaluated with HR and NHEJ reporters that are kindly provided by Dr. Gorbunova (Department of Biology, University of Rochester) [25,26]. The reporter cassette for detecting NHEJ or HR was digested with I-SceI to induce DSBs. The linearized reporter cassette of NHEJ or HR and DsRed were co-transfected into PC-3 cells after being treated with RD or vehicle for 24h. Cells were analyzed on the FACScalibur machine (Becton Dickinson, USA) using a green-versus-red fluorescent plot. Data were analyzed with the Cell Quest software (BD Biosciences).

### Assessment of anti-tumor effect of RD *in vivo*

To evaluate the *in vivo* effect of RD on PCa, 6-week-old male BALB/c-nu mice (Vital River Laboratories, Beijing, China) were used. Mice were allowed to acclimate for 1 week after arrival. Tumor xenografts were established by injecting 5×10^6^ PC-3 cells into the right flanks of mice. When tumors were detectable, the mice were randomized into treatment and control groups. Initial dosing was given at the time of pair matching (day 1). RD (30 mg/kg) or placebo (equivalent amounts of solvent) was given every second day by i.p. injection. Mice were monitored daily following treatments, and tumors were measured every second day by determining two perpendicular dimensions [length (L) and width (W)] using vernier calipers and calculated using the formula V=L×W^2^/2. After 20 days treatment, all mice were euthanized by air-ether anesthesia and their tumors resected for tumor mass measurement. Tumor tissues were fixed in 4% paraformaldehyde and used for TUNEL analysis (Calbiochem), immunoblotting, and immunofluorescence. All animal experiments were approved by the Ethics Committee of Shandong University School of Medicine (Permit Number: 2010038) and conducted accordingly.

### Statistical analysis

The data are presented as the mean ± S.D. of at least three independent experiments. The statistical significance of mean difference between the control and treated groups was determined by a paired t-test where *P* < 0.05 was considered statistically significant.

## Results

### RD induces cell cycle arrest and apoptosis in PC-3 cells

**Figure 1:**
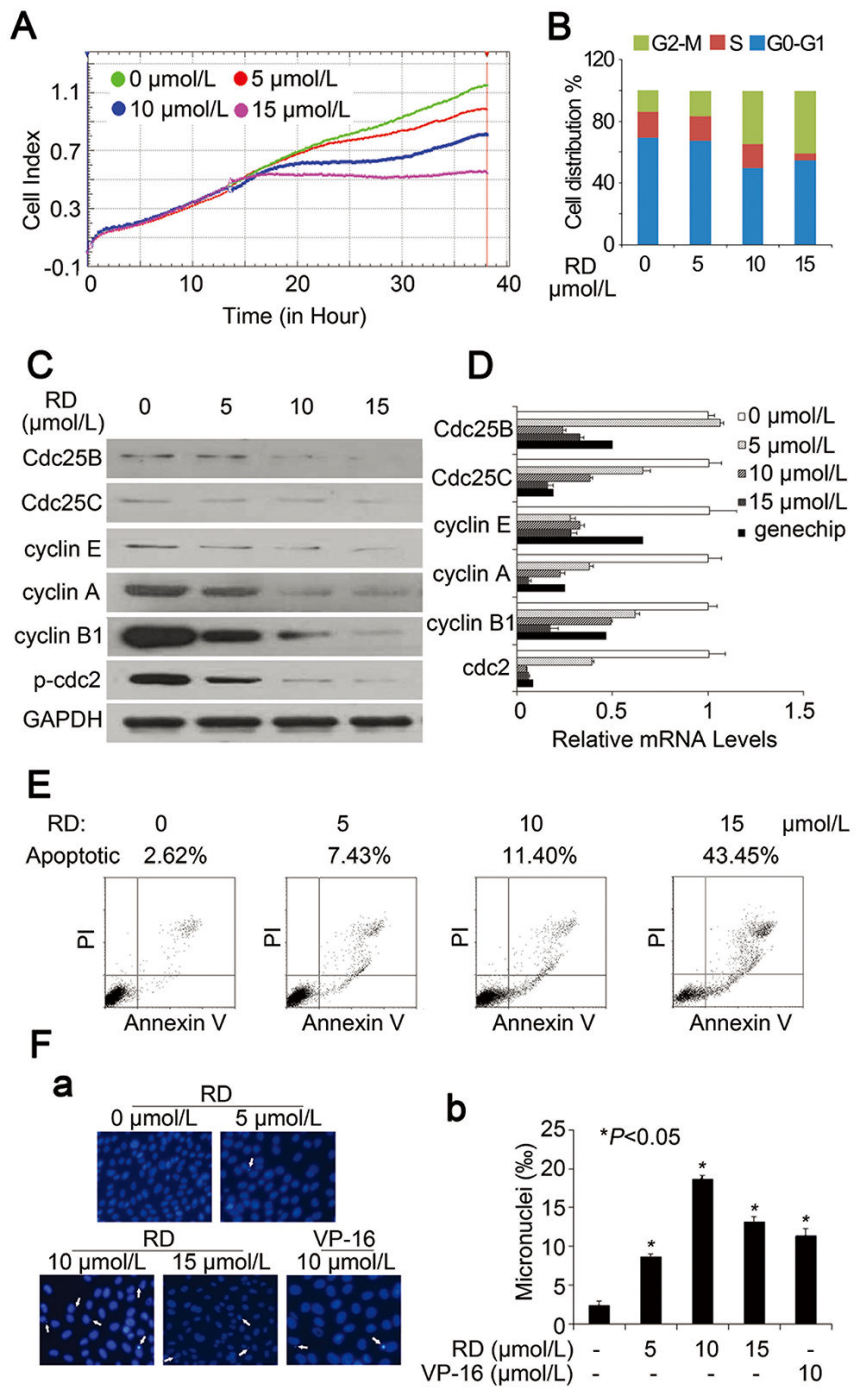
Effects of RD on cell proliferation, cell cycle, and apoptosis in PC-3 cells. *A*, Cell proliferation in response to RD was monitored by the cell index (CI) values using xCELLigence System. *B*, After exposure of PC-3 cells to RD for 24 h, cell cycle was analyzed by ﬂow cytometry. *C*, Immunoblot analysis of cell cycle regulators in cells treated with RD. *D*, mRNA levels of G2/M phase-related cycle regulators in RD-treated cells were determined by real-time PCR. Expression changes of those genes following RD treatment (10µmol/L) were also included. *E*, Percentage of apoptotic cells was analyzed by ﬂow cytometry. *F*, *a*, analysis of micronuclei formations in PC-3 cells treated with RD. *b*, the frequency of PC-3 cells containing micronuclei treated with RD or VP-16 for 24h. bars, SD. *, P < 0.05, significant difference from untreated group.

Since RD has been found to induce apoptosis in leukemia cells and lung cancer cells [16,18], we initiated our study to determine whether RD also exerted cytotoxic effect on PCa cells by continuously monitoring cell proliferation. As shown in Figure 1A, exposure of PC-3 cells to RD caused markedly suppression of cell proliferation in a dose- and time-dependent manner.

After 24h treatment with RD, cell cycle was significantly arrested in G2/M phase with increasing concentrations. Accordingly, RD treatment resulted in dose-dependent decreases in the expression of cyclin E, A, and B, which are the molecular markers correlated with G2/M phase. The family of Cdc25 phosphatases and the downstream target Cdc2 play an important role in S and G2-M progression. We also observed decreases in the expressions of Cdc25B, Cdc25C, and phosphor-Cdc2^Tyr15^ (inactive form) in a dose-dependent manner in treated cells (Figure 1C). qPCR assays showed that the expression of the Cdc2 at the transcript level was markedly decreased in RD-treated cells (Figure 1D), suggesting that RD suppressed the expression of Cdc2 at the mRNA level, which in turn led to a reduced phosphor-Cdc2. Meanwhile, changes in the mRNA expression of cyclin E, A, B, Cdc25B, and Cdc25C by RD (Figure 1D) were similar to the observations in Figure 1C, indicating that the pattern of cellular responses was consistent with the RD-induced perturbation of cell cycle in PC-3 cells. In addition to interference with cell cycle progression, RD was able to noticeably induce apoptosis in a dose-dependent manner (Figure 1E).

Microarray analysis revealed that genes related to damaged-DNA binding, DNA repair, cell cycle, and apoptosis were significantly changed in RD-treated cells when compared to the control as summarized in Table S1. Of those genes, we observed that cyclin E, A, B, Cdc2, Cdc25B, and Cdc25C were down-regulated dramatically in the RD-treated cells, consistent with results in Figure 1D.

### RD triggers DNA damage in PCa cells

We then determined whether exposure to RD at concentrations which were sufficient to induce cell cycle arrest and apoptosis resulted in DNA damage, because RD was able to inhibit Topo II in leukemia cells [16]. As expected, micronucleus formation was evident in cells treated with 5 µmol/L RD for 24h, and exhibited a dose-dependent increase (panel a in Figure 1F), similar to VP-16, a well-documented DNA-damage agent. The number of micronucleus in cells exposed to RD was summarized in panel b in Figure 1F. Therefore, these results suggested that RD induced DNA damage that was associated with the promotion of cell cycle arrest and apoptosis in PC-3 cells.

**Figure 2:**
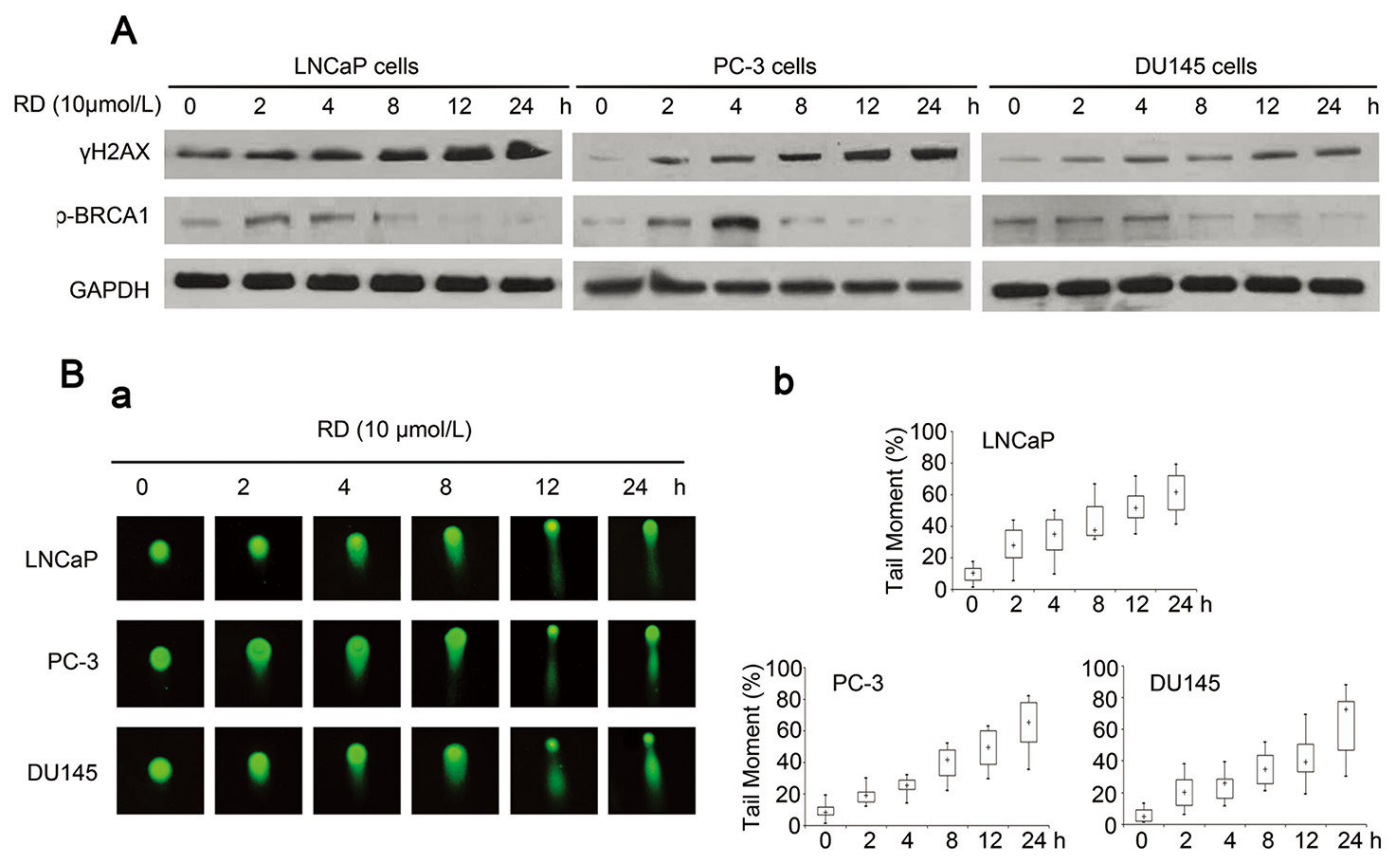
RD induced DSBs in PCa cells. *A*, Immunoblot analysis of expression levels of p-BRCA1, and γH2AX in LNCaP, PC-3, and DU145 cells exposed to RD, respectively. *B*, *a*, Neutral comet assay was performed to determine DNA fragment in RD-treated cells. *b*, Distribution of mean comet length (100 cells per sample) was calculated by box and whisker plot. Medians are indicated by cross; interquartile range (25-75%; IQRs) are indicated by open boxes. The whiskers are 1.5 times the IQR distribution.

To confirm the ability of RD in triggering DNA damage in PCa cells, we examined the changes of DNA damage markers by western blotting. As shown in Figure 2A, RD caused significant increases in the phosphorylation of histone H2AX^Ser139^ (γH2AX), a well-known DNA damage response indicator, at 2h and sustained up to 24h after treatment in both androgen-dependent (LNCaP) and androgen-independent PCa (PC-3 and DU145) cells. In terms of DNA damage response proteins, the expressions of phosphor-BRCA1 by RD were pronounced at early time-points and dropped down in cells after prolonged treatments (Figure 2A), suggesting that RD induced DNA damage response in PCa cells.

Furthermore, neutral comet assay was performed to test whether RD can induce DSBs in PCa cells. Results in Figure 2B showed that DNA tail moments in response to RD were detectable in cells as early as 2h treatment, and became more pronounced with prolonged treatment. Thus, the data indicated that RD significantly caused DSBs in PCa cells.

### RD affects ATM/ATR-dependent Chk1/Chk2 pathways in PC-3 cells

**Figure 3:**
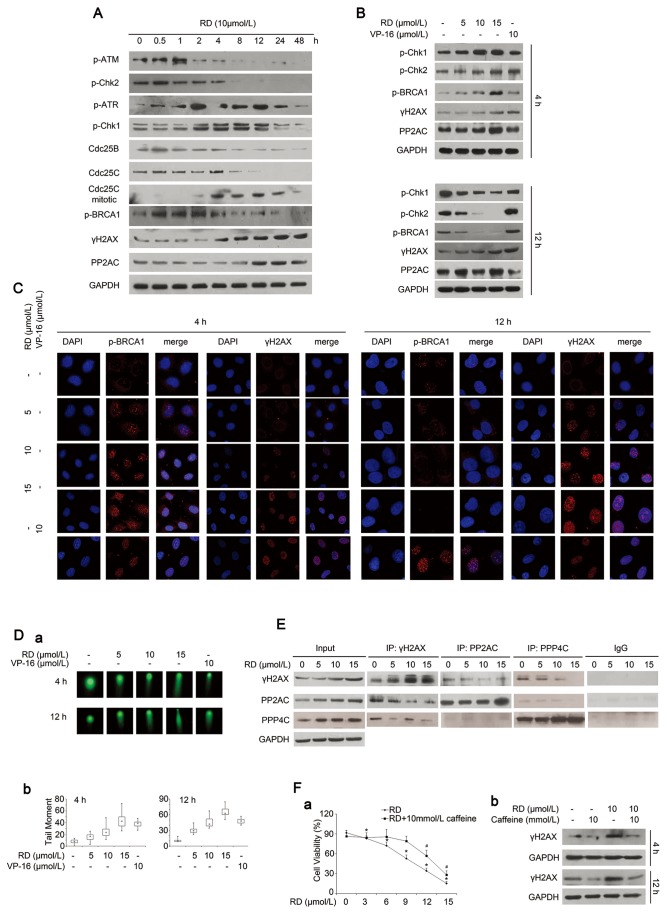
Effect of RD on DNA damage response signalings. *A*, Changes of DNA damage proteins in RD-treated cells were analyzed by western blotting. *B*, After treatment with chemicals for 4h or 12h, protein levels of DNA damage proteins were detected by western blotting. *C*, Immunoﬂuorescence staining of γH2AX foci and p-BRCA1 foci in PC-3 cells. *D*, *a*, Neutral comet assay of PC-3 cells treated with RD for 4h and 12h. *b*, Comet length was analyzed by box and whisker plot method (100 cells per sample). *E*, Associations of γH2AX, PP2AC, and PPP4C were determined by coimmunoprecipitation using anti-γH2AX, anti-PP2AC, anti-PPP4C, or normal IgG. *F*, PC-3 cells were pretreated with 10 mmol/L caffeine for 1h, and exposed to RD for 4h and 12h, *a*, cell viability measured by MTT assay; bars, SD. *, ^#^, P < 0.05, significant difference from control. *b*, changes of γH2AX were detected by western blotting.

To determine if ATM/ATR-Chk1/2 signaling pathways, which are well-identified as being activated following DNA damage, are involved in RD-induced DNA damage response, we first examined changes of factors known to be important for mediating ATM/ATR pathways. Kinetic studies displayed elevated phosphorylation of ATM and Chk2 (Thr^68^) was induced by RD as early as 30 min, but this phosphorylation level sharply declined afterwards. Whereas activation of ATR/Chk1 was observed at 2h treatment and persisted up to 24h as evidenced by accumulation of phosphor-ATR and phosphor-Chk1 (Ser^296^) in response to RD (Figure 3A). It should be noted that ATR/Chk1 was significantly activated by RD at the 2h treatment, where activation of ATM/Chk2 was impaired. Shifting activation of ATM to ATR suggested that other types of DNA lesions including replication interference and bulky lesions may also occur in addition to DSBs. Negative regulation of Cdc25 family members, downstream of Chk1/Chk2, is an important mechanism responsible for blocking mitotic entry after DNA damage [19]. As expected, downregulated Cdc25B/C and a pronounced induction of mitotic Cdc25C at 4h, which persisted following treatment, were observed in RD-treated cells when compared to the untreated cells (Figure 3A). An increase in the cleavage of PARP was also observed (Figure 3A).

DNA damage triggers a signaling cascade that leads to the formation of a repair complex at the breaks. We next assessed changes of protein BRCA1, a critical molecule in the initial recruitment of other repair proteins/enzymes at the breaks [20]. Activation of BRCA1 (phosphorylation at Ser^1524^) by RD was noted up to 4h and declined following treatment, which correlated well with the activation pattern of Chk2, suggesting Chk2 may actually phosphorylate BRCA1 in response to the damage (Figure 3A). Based on the observations above, we found that significant changes have occurred in the 4h and 12h treatments, both of which could be critical time points for RD-induced DNA damage response. Further studies (Figure 3B) displayed that, the pattern of the response involving γH2AX, phosphor-Chk1/2 and phosphor-BRCA1 at 4h and 12h was consistent with the observations in Figure 3A. These results were further supported by the observation in Figure 3C. As a control, Vp-16 was able to maintain elevated phosphor-Chk1/2, phosphor-BRCA1, and γH2AX levels after longer exposure when compared to those in RD treatments (Figure 3B and 3C), suggesting that different mechanisms contributed to the responses of RD and VP-16 treatments. In accordance with the alterations of DNA damage response proteins, pronounced comet tails were shown to present in cells exposed to RD (panels a and b in Figure 3D). Of note, elevated γH2AX that may be phosphorylated by ATM/ATR kinases [21,22] was evident at 4h and sustained up to 48h following RD treatment, where the activated-ATM/ATR by RD was abrogated (Figure 3A). We also analyzed changes of protein phosphatase 2A (PP2A) and protein phosphatase 4 (PP4), which are implicated in dephosphorylating γH2AX [23,24]. After 24h treatment, RD caused increased PP2AC (catalytic subunit of PP2A), and PPP4C (catalytic subunit of PP4) (Figure 3E), indicating that H2AX remained phosphorylated in the presence of elevated PP2AC and PPP4C. Co-immunoprecipitation results showed that γH2AX was markedly noticeable with progressively decreased PP2AC or PPP4C in complexes immunoprecipitated by anti-γH2AX, anti-PP2AC or anti-PPP4C antibodies (Figure 3E), suggesting that impaired associations of γH2AX/ PP2AC/PPP4C by RD may, at least in part, contribute to the substantial accumulation of γH2AX.

Additionally, caffeine, an inhibitor of ATM/ATR signaling, almost completely abrogated the ability of RD to promote H2AX phosphorylation during treatment, which was accompanied with the significant reversal of RD-induced cell death (Figure 3F). Together, the data clearly demonstrated that ATM/ATR-mediated cascade pathways played a crucial role in response to RD-induced DNA damage, leading to the promotion of cells to enter lethal mitosis.

### RD inhibits NHEJ and HR in PC-3 cells

**Figure 4:**
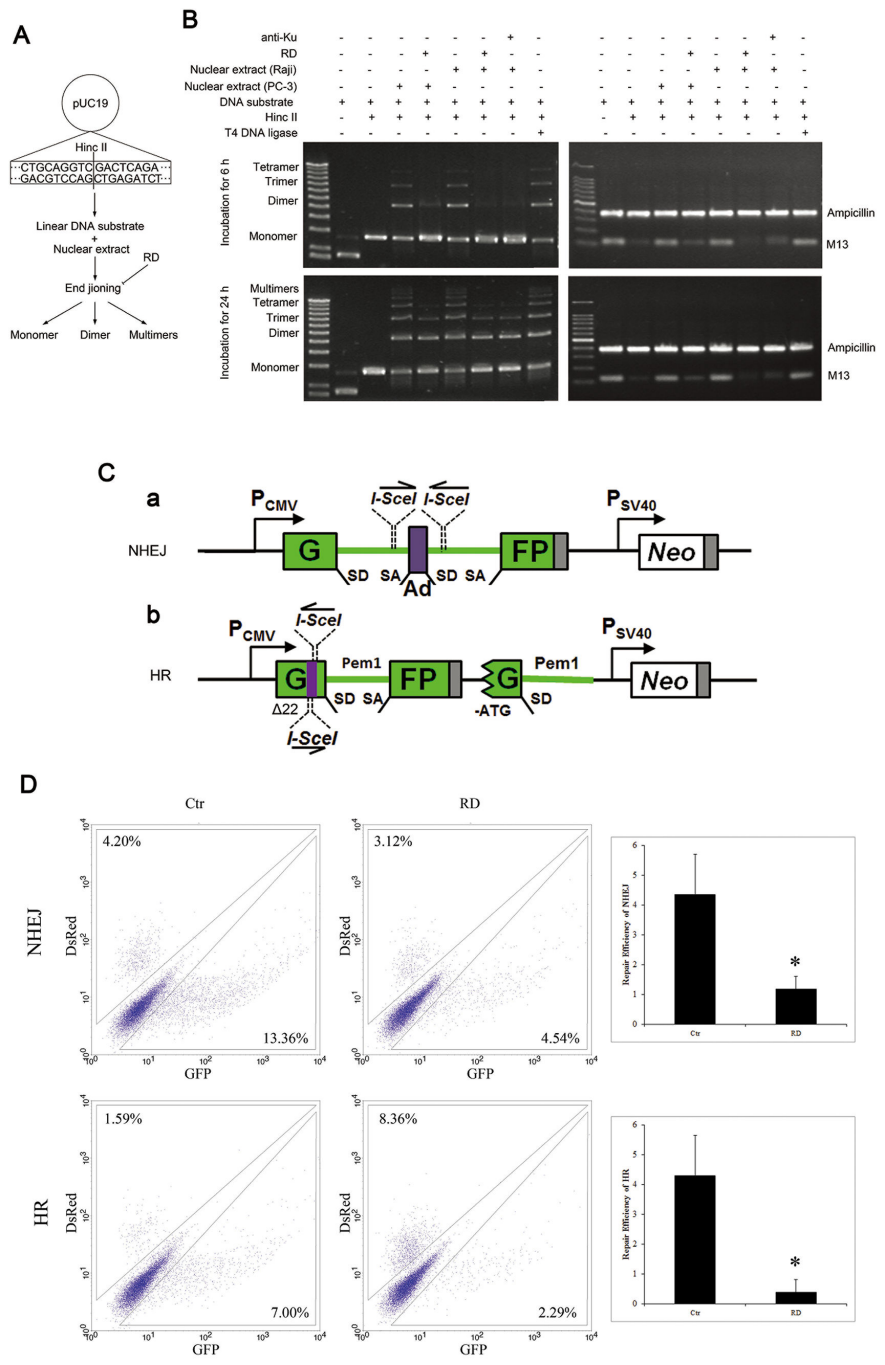
Effect of RD on activities of NHEJ and HR. *A*, Schematic diagram of the principle of DNA End-joining Assay. *B*, After being treated with RD, or with blocking antibodies Ku 70/86, the relative ability of NHEJ were determined. *C*, *a*, Schematic diagram of the principle of NHEJ assays. *b*, Schematic diagram on the principle of HR assays. *D*, The numbers of GFP^+^ and DsRed^+^ cells were determined by flow cytometry, and typical FACS traces are shown on the left. The ratio of GFP^+^ to DsRed^+^ cells was used as a measure of repair efficiency.

To determine the effects of RD on DSBs repair, we developed a cell-free DNA end-joining assay to evaluate the relative contribution of NHEJ in DNA end-joining [17]. Linearized plasmid pUC19 DNA by enzyme HincII was incubated with nuclear protein extracts, and end-joining activity was reflected by the appearance of linear dimers and multimers which were amplified by PCR with M13 primers flanking the end-joined junction (Figure 4A, 4B). After treatment with RD for 6h or 24h, NHEJ activity of the nuclear extract was markedly suppressed as indicated by appearance of a strong monomer band and correspondingly decreased multimer products, while dimer, trimer, and tetramer bands were evident in the control group (Figure 4B). As a positive control, NHEJ activity was also impaired by RD during incubation of the DNA substrate with Raji cell nuclear extract (Active Motif) under the same conditions (Figure 4B). In addition, incubation of linear DNA with blocking antibodies directed against Ku70 and Ku86 in nuclear proteins led to decreased multimer bands, similar to the observation in RD treatment (Figure 4B), providing evidence that Ku heterodimer Ku70/Ku86 are two of the important proteins in RD-mediated DSBs repair.

We went a further step to evaluate effects of RD on NHEJ and HR using *in vivo* assays as described by Dr. Gorbunova [25,26]. For detection of NHEJ activity, the reporter GFP will be produced when NHEJ is active to repair the DSBs induced by enzyme digestion (Figure 4C, a). For detection of HR, successful gene conversion events can reconstitute active GFP gene by repairing enzyme-produced DSBs (Figure 4C, b). After being treated with RD for 24h, PC-3 cells were co-transfected with enzyme I-SceI-digested reporter cassette of NHEJ or HR, and DsRed plasmid to normalize transfection efficiency. Repair of I-SceI-induced breaks will result in the appearance of GFP^+^ cells [26]. After transfection, cells were analyzed by flow cytometry, and the ratio between GFP^+^ and DsRed^+^ cells was used as a measure of HR or NHEJ efficiency. As shown in Figure 4D, the GFP signal significantly declined in either NHEJ or HR repair systems in cells treated with RD, indicating that DSBs repair was impaired in response to RD. Together, the data demonstrated that RD was able to inhibit NHEJ and HR, and suppressed DSBs repair in PC-3 cells.

### RD downregulates DNA repair proteins in PC-3 cells

**Figure 5:**
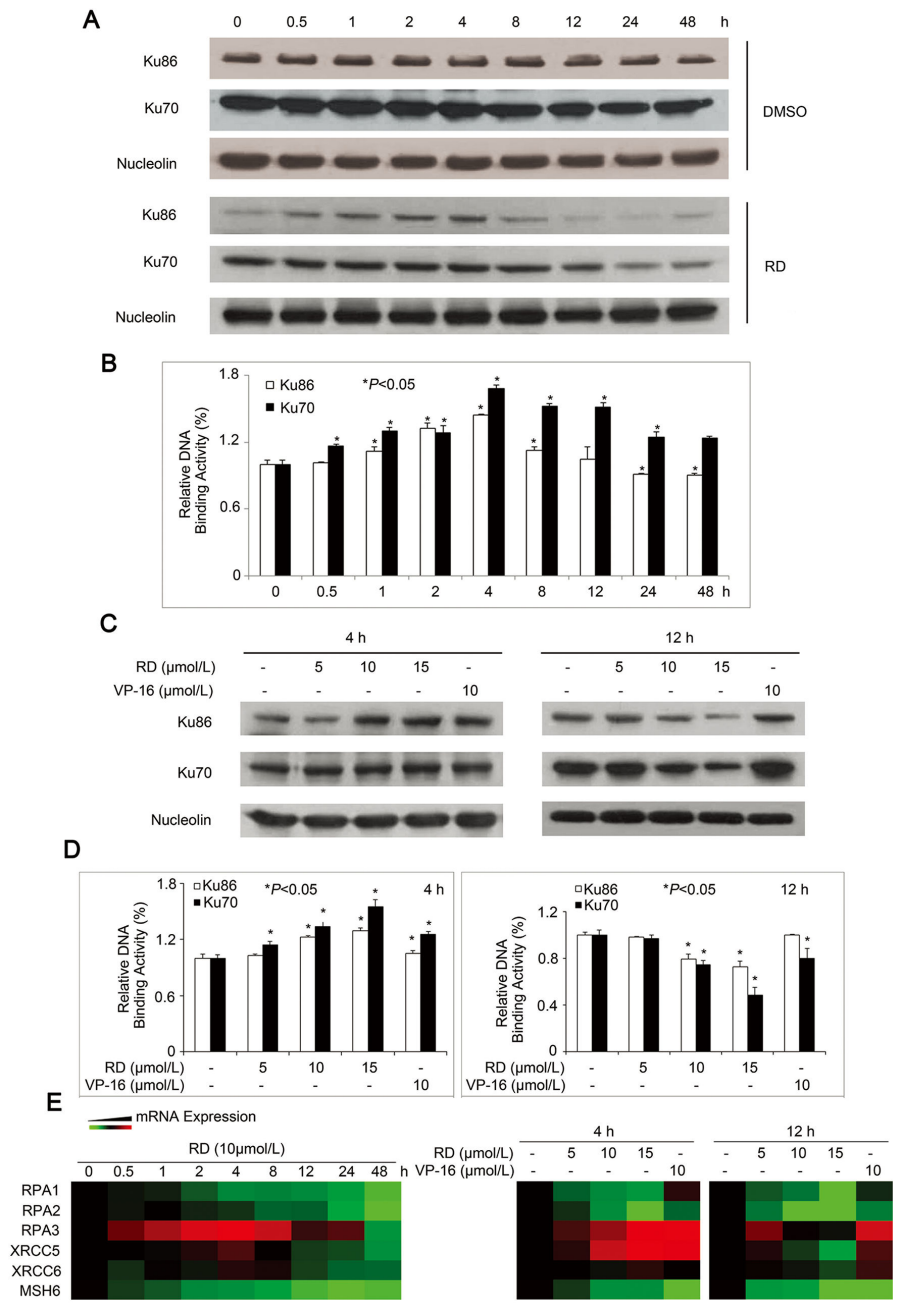
Effect of RD on DNA repair in PC-3 cells. *A* and *C*, Analysis of the expressions of Ku70 and Ku86 in nuclear proteins by western blot assay. *B* and *D*, Analysis of DNA end-binding activity of Ku70 or Ku86 in PC-3 cells exposed to RD. *, P < 0.05, significant difference from control. *E*, Heatmap for mRNA levels of DNA repair genes in RD-treated cells which were determined by real-time RT-PCR. Red: over-expression, green: under-expression, black: unchanged expression, gray: no detection.

Based on the observations above, we further clarified the role of Ku70/Ku86 in response to RD-induced DNA damage. After incubation of cells with RD for different time periods, Ku86 increased and peaked at 4h, then decreased rapidly up to 48h, while Ku70 remained unchanged until 12h and declined after that (Figure 5A). DNA end-binding activity of Ku70/Ku86 displayed that, compared with the untreated cells, the binding activities of both Ku70/Ku86 in treated cells were steadily enhanced up to 4h and then decreased during the rest of the exposure period (Figure 5B), consistent with the results in Figure 5A. Additionally, we confirmed the dose-dependent inhibitory effect of RD on the expression and binding activity of Ku70/Ku86 at 4h and 12h treatments (Figure 5C, 5D). Moreover, qPCR assays demonstrated that DNA repair associated *RPA1-3*, *XRCC5*, *XRCC6*, and *MSH6* were downregulated by RD (Figure 5E). Together, these observations indicated that RD impaired DNA repair in response to DNA damage.

### RD triggers apoptosis associated with the induction of DNA damage in PC-3 xenograft

**Figure 6:**
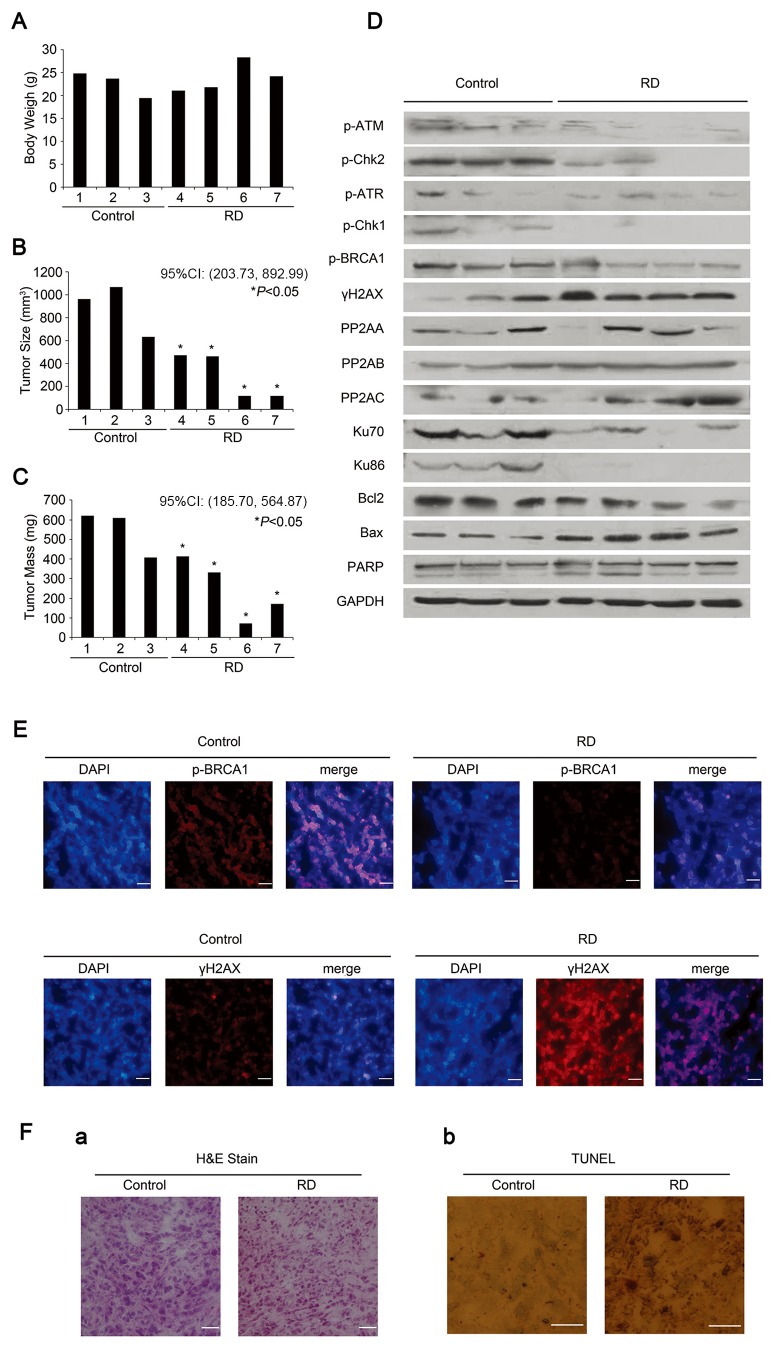
RD inhibits tumor growth in xenograft mice. *A*-*C*, Effect of RD on tumor growth and body weight of nude mice. *D*, western blot analysis of DNA damage and repair, and apoptosis proteins from tumor tissues. *E*, immunofluorescence staining of p-BRCA1 and γH2AX in RD-treated and placebo tissues. Nuclei were stained with DAPI. Scale bars, 100 µm. *F*, *a*, H&E staining of tissues from RD- and placebo-treated nude mice. *b*, Tissues were fixed and stained by TUNEL followed by DAB staining. Nuclei were counterstained with methyl green. Brown stain around green nuclei indicates apoptotic cells.

To determine whether RD could induce tumor cell apoptosis via induction of DNA damage *in vivo*, as observed in cultured cells, human PC-3 xenografts were developed in male nude mice. Administration of RD had no significant effect on either initial or ﬁnal body weight in tumor-bearing mice compared to placebo group (Figure 6A). After 20d treatments, tumors arising from control animals resulted in killing of two animals during the experimental period, whereas tumors from RD-treated mice had an significantly smaller size (Figure 6B), indicating significant tumor growth inhibition by RD. Reduction of tumor mass in RD treated-mice was also observed (Figure 6C). Changes of molecular markers associated with DNA damage in treated mice displayed that RD caused DNA-damage response via ATM/ATR-mediated signaling as evidenced by substantial accumulation of γH2AX, abrogation of phosphor-BRCA1, and Ku70/ Ku86 abundance (Figure 6D and 6E), consistent with the results in culture cells. Meanwhile, RD-mediated alterations in the expressions of the PP2A family members were similar to the observations shown in culture cells. Additionally, compared with placebo control, abrogation of Bcl-2, accumulation of Bax, and cleavage of PARP were evident in RD-treated mice (Figure 6D). TUNEL assay also supported the observations that apoptotic cells were pronounced in RD-treated mice (Figure 6F a and b). The data demonstrated that RD triggered DNA damage and impaired repair proteins, leading to apoptotic cell death, which contributed to its antitumor effect.

## Discussion

Here we presented for the first time that RD induced cell cycle arrest at G2/M phase and apoptosis, and that one of the plausible mechanisms accounting for its antitumor activity occurred through the activation of ATM/ATR-dependent DNA damage response and inhibition of NHEJ and HR repair, subsequently leading cells to enter mitotic cell death.

In response to DNA-damaging agents, the checkpoint functions of Chk1 and Chk2 are activated by ATR/ATM signaling [27,28]. Our data demonstrated that RD predominantly initiated the activation of ATM at an early time with subsequent onset of a robust activation of ATR after the phosphor-ATM dropped down during treatment, leading to changes in the phosphor-Chk1^Ser296^ and phosphor-Chk2^Thr68^ correspondingly. This suggests that RD may initially cause DSBs, and its prolonged exposure resulted in bulky DNA lesions, including SSBs and other lesions that contribute to its cytotoxicity. Regarding whether DNA damage agents can activate ATM or ATR or both, it would depend on the type of agents and cell types with different cellular contexts. For example, VP-16 elicits primarily ATR activation [29,30], however, camptothecin activates either ATM or ATR in DNA damage events in different cancer cell lines [14], to some extent, was similar to RD in PCa cells. The detailed mechanism by which differential activation of ATM/ATR by RD also remains to be clarified in the future investigation.

Activation of ATM/ATR can be specifically analyzed by detection of γH2AX. In response to RD, the appearance of long-lasting γH2AX was evident even though ATM/ATR levels significantly decreased after prolonged treatment. This could be the combined result of a persistent cell cycle arrest in the absence of efficient DNA repair. Defect in the repair of DNA damage has been observed in PCa cells, resulting in malignant cells with a weak capacity for DNA repair [31,32]. Each type of DNA damage elicits a specific cellular repair response [33]. RPA proteins bind directly to single stranded DNA where it organizes and protects ssDNA during DNA replication, recombination and repair. Ku protein heterodimer Ku70/86 is critical for the repair of dsDNA breaks. The G/T binding protein (MSH6) is a mismatch repair (MMR) protein which specifically recognizes mismatched G/T base pairs in dsDNA where it triggers excision and repair. We found RD exhibited extensive inhibitory effects on these DNA repair proteins/enzymes (Figure 5E). However, XRCC5, also known as Ku86, is activated after very short-term RD treatment and then dropped down significantly during long exposure both at mRNA and protein levels, suggesting that RD may have a regulatory effect on the expression of XRCC5 at transcriptional level, and need to be investigated. Unlike other DNA repair enzymes which had been continuously suppressed, activation of RPA3 mRNA was observed at 0.5h after RD-treatment and persisted up to 24h, suggesting that both DSB- and SSB-associated mechanisms were involved in RD-triggered DNA damage in PC-3 cells, and stalled replication forks and bulky lesions may also occur. It has been demonstrated that the ATRIP–RPA–ssDNA interaction is essential for ATR activation [34]. In our study, the pattern of changes of RPA3 was similar to that of ATR, as indicated that strong phosphorylation levels of ATR were also increased at 0.5h and became robust for up to 24h RD-treatment, suggesting that the activation of ATR in response to RD was, at least in part, related to the expression of RPA3. Identification of the roles of RPA3 and XRCC5 in RD-triggered DNA damage remains to be addressed in future study.

In response to DNA damage, cells with damaged DNA could undergo apoptosis if damaged-DNA is hardly to be repaired. An exciting finding of our study is that RD inhibited DNA repair in addition to DNA damage induction, and induced apoptosis in PCa cells *in vitro* and *in vivo*.

In conclusion, this study presented a mechanistic basis for antitumor effect of RD in PCa cell and animal models through induction of DNA damage, inhibition of DNA repair activity, and G2/M phase arrest involving ATM/ATR/Chk1/2 pathways. This finding provides the foundation for the use of RD as a chemotheraputic candidate.

## Supporting Information

Table S1 **Gene expression changes in PC-3 cells treated with 10 µmol/L Riccardin D for 24h.**
(DOCX)
