# Supplementary material for: Riccardin D Exerts Its Antitumor Activity by Inducing DNA Damage in PC-3 Prostate Cancer Cells In Vitro and In Vivo
Source: PLoS One. 2013 Sep 17;8(9):e74387. doi: 10.1371/journal.pone.0074387 (PMC3775815; doi:10.1371/journal.pone.0074387)
Supplement: Table S1 — Gene expression changes in PC-3 cells treated with 10 µmol/L Riccardin D for 24h. (DOCX) [file pone.0074387.s001.docx]

| Table S1.Gene expression changes in PC-3 cells treated with 10 μmol/L Riccardin D for 24h | | | | | |
| --- | --- | --- | --- | --- | --- |
| Gene Symbol | Probe Set ID | GeneBank | Gene Function | Regulation | Change |
| ANKRD17 | 225852_at | BE463523 | Mismatch Repair | I | 1.3 |
| APEX2 | 204408_at | NM_014481 | Other Genes Involved in DNA Repair | D | 0.6 |
| ATM | 212672_at | U82828 | Other Genes Involved in DNA Repair | I | 2.1 |
| ATRX | 208861_s_at | U72937 | Other Genes Involved in DNA Repair | I | 1.2 |
| BRCA1 | 204531_s_at | NM_007295 | Damaged DNA Binding | D | 0.5 |
| CDC25A | 204695_at | AI343459 | Cell Cycle Arrest | D | 0.2 |
| CDC25B | 201853_s_at | NM_021873 | Cell Cycle Arrest | D | 0.5 |
| CDC25C | 205167_s_at | NM_001790 | Cell Cycle Arrest | D | 0.2 |
| CDK7 | 211297_s_at | L20320 | Other Genes Involved in DNA Repair | I | 1.5 |
| CHEK1 | 238075_at | AA224205 | Cell Cycle Arrest | D | 0.5 |
| DDB2 | 203409_at | NM_000107 | Damaged DNA Binding | D | 0.4 |
| DDIT3 | 209383_at | BC003637 | Cell Cycle Arrest | I | 6.1 |
| DDIT4 | 202887_s_at | NM_019058 | Apoptosis Genes | I | 3.5 |
| ERCC1 | 228131_at | BG111047 | Damaged DNA Binding | D | 0.3 |
| ERCC2 | 213468_at | AI918117 | Other Genes Involved in DNA Repair | D | 0.6 |
| ERCC3 | 202176_at | NM_000122 | Damaged DNA Binding | D | 0.7 |
| ERCC4 | 235215_at | AI694544 | Other Genes Involved in DNA Repair | I | 1.5 |
| ERCC5 | 202414_at | NM_000123 | Other Genes Involved in DNA Repair | I | 1.2 |
| EXO1 | 204603_at | NM_003686 | Double-strand Break Repair | D | 0.3 |
| FANCG | 203564_at | NM_004629 | Damaged DNA Binding | D | 0.5 |
| FEN1 | 204768_s_at | NM_004111 | Double-strand Break Repair | D | 0.3 |
| GADD45A | 203725_at | NM_001924 | Apoptosis Genes | I | 4.3 |
| GTF2H1 | 202453_s_at | NM_005316 | Other Genes Involved in DNA Repair | I | 1.5 |
| GTSE1 | 204318_s_at | NM_016426 | Cell Cycle Arrest | D | 0.3 |
| H2AFX | 212525_s_at | AA760862 | Damaged DNA Binding | D | 0.4 |
| HUS1 | 204884_s_at | NM_004507 | Cell Cycle Arrest | I | 1.6 |
| LIG1 | 202726_at | NM_000234 | Other Genes Involved in DNA Repair | D | 0.2 |
| LIG4 | 227766_at | AI829314 | Other Genes Involved in DNA Repair | I | 1.9 |
| MAPK12 | 206106_at | AL022328 | Cell Cycle Arrest | MD | 0.6 |
| MBD4 | 209580_s_at | AF114784 | Base-excision Repair | I | 1.7 |
| MLH1 | 202520_s_at | NM_000249 | Mismatch Repair | I | 1.1 |
| MLH3 | 204838_s_at | NM_014381 | Mismatch Repair | I | 2.0 |
| MRE11A | 242456_at | AA931565 | Double-strand Break Repair | D | 0.4 |
| MSH2 | 209421_at | U04045 | Mismatch Repair | D | 0.6 |
| MSH6 | 211450_s_at | D89646 | Mismatch Repair | D | 0.7 |
| N4BP2 | 228242_at | BF055201 | Mismatch Repair | I | 4.6 |
| NBN | 202906_s_at | AF049895 | Double-strand Break Repair | I | 1.2 |
| NTHL1 | 209731_at | U79718 | Base-excision Repair | D | 0.3 |
| NUDT1 | 204766_s_at | NM_002452 | Other Genes Involved in DNA Repair | D | 0.1 |
| OGG1 | 205760_s_at | NM_016821 | Base-excision Repair | D | 0.7 |
| PCNA | 201202_at | NM_002592 | Other Genes Involved in DNA Repair | D | 0.3 |
| AIFM1 | 205512_s_at | NM_004208 | Apoptosis Genes | D | 0.8 |
| PMS1 | 213677_s_at | BG434893 | Double-strand Break Repair | I | 2.1 |
| PMS2 | 221206_at | NM_024521 | Double-strand Break Repair | I | 1.2 |
| PPP1R15A | 202014_at | NM_014330 | Apoptosis Genes | I | 8.0 |
| PRKDC | 210543_s_at | U34994 | Double-strand Break Repair | D | 0.7 |
| RAD17 | 207405_s_at | NM_002873 | Cell Cycle Arrest | I | 1.4 |
| RAD21 | 200607_s_at | BG289967 | Double-strand Break Repair | MD | 0.7 |
| RAD50 | 208393_s_at | NM_005732 | Double-strand Break Repair | I | 2.0 |
| RAD51 | 205024_s_at | NM_002875 | Damaged DNA Binding | D | 0.4 |
| RAD51C | 206066_s_at | NM_002876 | Damaged DNA Binding | D | 0.5 |
| RAD54L | 204558_at | NM_003579 | Other Genes Involved in DNA Repair | D | 0.3 |
| RPA1 | 201528_at | BG398414 | Other Genes Involved in DNA Repair | D | 0.4 |
| RPA2 | 201756_at | NM_002946 | Other Genes Involved in DNA Repair | D | 0.5 |
| RPA3 | 209507_at | BC005264 | Other Genes Involved in DNA Repair | D | 0.3 |
| SMC1A | 201589_at | D80000 | Cell Cycle Arrest | D | 0.6 |
| SUMO1 | 208762_at | U83117 | Other Genes Involved in DNA Repair | D | 0.8 |
| TREX2 | 213334_x_at | BE676218 | Other Genes Involved in DNA Repair | D | 0.4 |
| UNG | 202330_s_at | NM_003362 | Base-excision Repair | D | 0.4 |
| CCNO | 210021_s_at | BC004877 | Base-excision Repair | I | 2.6 |
| XRCC3 | 216299_s_at | AK022829 | Damaged DNA Binding | D | 0.0 |
| XRCC4 | 210813_s_at | BC005259 | Double-strand Break Repair | D | 0.7 |
| XRCC5 | 208643_s_at | J04977 | Double-strand Break Repair | D | 0.5 |
| XRCC6 | 200792_at | NM_001469 | Double-strand Break Repair | D | 0.7 |
| XRCC6BP1 | 227678_at | AI628122 | Double-strand Break Repair | D | 0.4 |
| ZAK | 218833_at | NM_016653 | Cell Cycle Arrest | I | 2.0 |
|  |  |  | I = Increase, D = Down, MD = Moderate Down | | |
